# Recruitment of BAD by the Chlamydia trachomatis Vacuole Correlates with Host-Cell Survival

**DOI:** 10.1371/journal.ppat.0020045

**Published:** 2006-05-19

**Authors:** Philippe Verbeke, Lynn Welter-Stahl, Songmin Ying, Jon Hansen, Georg Häcker, Toni Darville, David M Ojcius

**Affiliations:** 1 Institut Jacques Monod, Université Paris—Denis Diderot, Paris, France; 2 Institute for Medical Microbiology, Technische Universität München, Munich, Germany; 3 Department of Microbiology and Immunology, University of Arkansas for Medical Sciences, Little Rock, Arkansas, United States of America; 4 School of Natural Sciences, University of California Merced, Merced, California, United States of America; University of California San Francisco, United States of America

## Abstract

Chlamydiae replicate intracellularly in a vacuole called an inclusion. Chlamydial-infected host cells are protected from mitochondrion-dependent apoptosis, partly due to degradation of BH3-only proteins. The host-cell adapter protein 14-3-3β can interact with host-cell apoptotic signaling pathways in a phosphorylation-dependent manner. In *Chlamydia trachomatis-*infected cells, 14-3-3β co-localizes to the inclusion via direct interaction with a *C. trachomatis-*encoded inclusion membrane protein. We therefore explored the possibility that the phosphatidylinositol-3 kinase (PI3K) pathway may contribute to resistance of infected cells to apoptosis. We found that inhibition of PI3K renders C. trachomatis-infected cells sensitive to staurosporine-induced apoptosis, which is accompanied by mitochondrial cytochrome *c* release. 14-3-3β does not associate with the Chlamydia pneumoniae inclusion, and inhibition of PI3K does not affect protection against apoptosis of C. pneumoniae-infected cells. In *C. trachomatis-*infected cells, the PI3K pathway activates AKT/protein kinase B, which leads to maintenance of the pro-apoptotic protein BAD in a phosphorylated state. Phosphorylated BAD is sequestered via 14-3-3β to the inclusion, but it is released when PI3K is inhibited. Depletion of AKT through short-interfering RNA reverses the resistance to apoptosis of C. trachomatis-infected cells. BAD phosphorylation is not maintained and it is not recruited to the inclusion of *Chlamydia muridarum,* which protects poorly against apoptosis. Thus, sequestration of BAD away from mitochondria provides C. trachomatis with a mechanism to protect the host cell from apoptosis via the interaction of a *C. trachomatis-*encoded inclusion protein with a host-cell phosphoserine-binding protein.

## Synopsis

Chlamydia trachomatis is the most common cause of sexually transmitted bacterial infections in humans. These bacteria survive and replicate within a vacuole in the infected cell called an inclusion, producing up to a thousand bacteria per inclusion within a day of infection. Despite the large size of the inclusion, the infected cell survives long enough for the pathogens to complete their infection cycle and then infect new host cells. The researchers describe a novel mechanism for protection of the host cell by *Chlamydia,* namely activation of enzymes involved in host-cell survival. These enzymes, called kinases, cause the phosphorylation and inactivation of a protein, BAD, which can promote “cell suicide” (apoptosis) of uninfected cells. BAD phosphorylation is accompanied by recruitment of BAD to the chlamydial inclusion, where BAD binds to a cellular adapter protein, 14-3-3β. The adapter protein, in turn, is attracted to the inclusion by a membrane protein produced by *Chlamydia.* Thus, the chlamydial inclusion sequesters BAD away from mitochondria, where BAD could induce host-cell apoptosis. The cross talk between chlamydiae and host-cell signaling pathways demonstrates a novel mechanism of pathogen modulation of host-cell biology, and represents a potential therapeutic target for blocking infection by this prevalent pathogen.

## Introduction

Chlamydia trachomatis is the leading cause of bacterial sexually transmitted diseases worldwide; strains of C. trachomatis responsible for ocular infections lead to blindness, while Chlamydia pneumoniae causes pneumonia in humans and may accelerate atherosclerosis [1–6].

All *Chlamydia* species are intracellular pathogens that primarily infect epithelial mucosa [7]. Chlamydiae spend most of their developmental cycle within a membrane-bound vacuole, or inclusion, which avoids fusion with host-cell lysosomes. Despite their intravacuolar localization, the bacteria interact with the host cell to ensure that their inclusion remains a safe haven for replication [8], but at the same time allows for acquisition of essential nutrients from the host-cell cytosol [9].

Many membrane-trafficking and metabolic changes in the host cell are thought to be controlled by chlamydial proteins that are inserted into the inclusion membrane or secreted into the host cytosol. Similar to other Gram-negative bacterial pathogens, *Chlamydia* species possess a specialized type III secretion apparatus that behaves as a molecular syringe, enabling the bacteria to inject virulence factors from the bacteria into the host cytosol [10,11]. The metabolic demands made on the host cell by the growing inclusion [9,12] may activate a number of stress-related signaling pathways, including mitogen-activated protein kinases (MAP kinases or ERKs) [13–16]. Significantly, many stress-responsive pathways are involved in adaptation to environmental changes and affect apoptosis.

Apoptosis is required for the development of metazoans and is crucial for the maintenance of cellular homeostasis in mammals [17]. Microbial pathogens can inhibit or stimulate apoptosis of the infected cell [18,19]. In some cases, infection can result in both induction and inhibition of apoptosis at different stages of the infection cycle [20]. Likewise, it has been proposed that chlamydiae may induce cell death at the end of the infection cycle, but inhibit apoptosis at the beginning of the cycle [21].

The first reports of an anti-apoptotic effect of chlamydial infection showed that infection of epithelial cells and macrophages with C. trachomatis or C. pneumoniae protects against externally applied insults that induce apoptosis in uninfected cells [22–25]. These studies demonstrated that chlamydial infection prevents the release of cytochrome *c* from mitochondria and subsequent activation of caspases, suggesting that the apoptotic pathway may be blocked upstream from mitochondria. This view was reinforced by the observation that *Chlamydia*-infected cells are resistant to apoptotic pathways that rely on a mitochondrial amplification step, but not when caspases are activated directly [26]. More recently, it was shown that part of the resistance to apoptosis is due to degradation in infected cells of pro-apoptotic BH3-only proteins, including BIM, PUMA, and BAD [27–29]. These mediators lie upstream of mitochondria, where they activate BAX and BAK, which oligomerize on mitochondria and stimulate cytochrome *c* release [30].

Nonetheless, the possibility that stress-dependent or other signal transduction pathways, in parallel with BH3-only protein degradation, may contribute to protection against apoptosis has not been investigated. Importantly, the chlamydial inclusion membrane contains abundant transmembrane proteins encoded by the chlamydial genome, named Inc proteins, which participate in the development of the chlamydial inclusion [31]. A yeast two-hybrid analysis with one of the proteins, IncG, has shown that it interacts with a host cell protein, 14-3-3β [32]. This adapter protein is normally distributed in the cytosol, but in C. trachomatis-infected cells, it interacts and co-localizes with IncG [32]. The function for the IncG/14-3-3β interaction was not elucidated, but in uninfected cells, 14-3-3β can interact with several host-cell signaling pathways in a phosphorylation-dependent manner, including those involved in cell-cycle regulation, membrane trafficking, and apoptosis. The 14-3-3 isoforms can form homo- and heterodimers both in vivo and in vitro, which is thought to facilitate their interaction with different components of signaling pathways [33,34]. Significantly, in the presence of survival factors, cytosolic 14-3-3 proteins sequester phosphorylated BAD away from its targets in the mitochondria [35,36]. In the absence of survival factors, BAD is dephosphorylated and associates with mitochondria, resulting in release of cytochrome *c* and cell death. BAD itself is phosphorylated by AKT/protein kinase B (PKB), which is activated in the presence of survival factors by phosphatidylinositol-3 kinase (PI3K) [35–37].

The goal of this study was therefore to determine whether stress-induced MAP kinase pathways or PI3K may contribute to resistance of C. trachomatis-infected cells to apoptosis. We found that infection leads to activation of the PI3K pathway, which results in phosphorylation of BAD and its recruitment to chlamydial inclusions that express IncG. As our results revealed a predominant role for the PI3K pathway, we evaluated involvement of AKT through RNA interference and found that AKT plays an essential role in this protection. Our results further indicate that the PI3K pathway plays no role in protection against apoptosis in the absence of BAD phosphorylation or during infection with *C. pneumoniae,* which does not express IncG.

## Results

### The Lymphogranuloma Venereum Strain of C. trachomatis Protects Infected Cells against Staurosporine-Induced Apoptosis through a PI3K-Dependent Pathway

**Figure 1:**
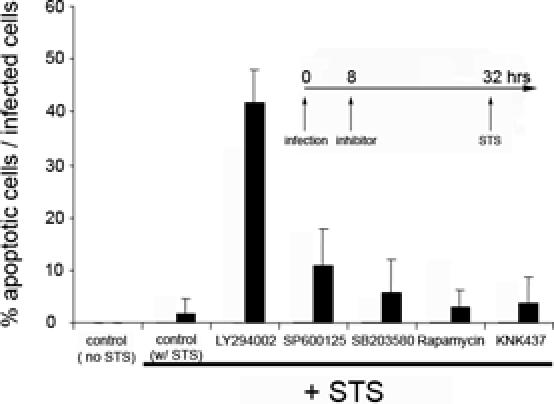
Signal-Transduction Pathways Involved in Resistance of C. trachomatis-Infected Cells against Apoptosis All cells were infected with C. trachomatis at an MOI of 1.0 for 32 h and then treated overnight with 2 μM STS. In uninfected cells that had been treated with STS under the same conditions, 83 ± 10% of the cells had apoptotic nuclei. Inhibitors were added 24 h before STS treatment, as indicated in the inset. The inhibitor concentrations (and targeted mediators) were: 50 μM LY294002 (PI3K), 10 μM SB203580 (P38), 10 μM U0126 (MEK5), 10 μM SP600125 (JNK1/2), 5 nM rapamycin (mTOR), and 50 μM KNK437 (heat shock proteins). The “control (no STS)” contained infected cells, while “control w/STS” contained infected cells that were incubated with buffer without inhibitor and then treated with STS. DNA was revealed by Hoechst staining, and values are expressed as the % of infected cells that had apoptotic nuclei. The average and SD were calculated from the values obtained from at least 50 microscope fields. The experiment was performed on three separate days, and results from a representative experiment are shown.

As infection of HeLa cells with the lymphogranuloma venereum (LGV)/L2 serovar has been reported to inhibit apoptosis in infected cells [22,26,27], we first measured the response of C. trachomatis-infected epithelial cells to treatment with the apoptosis inducer, staurosporine (STS). Thus, cells were infected with the LGV/L2 serovar and then treated with STS before immunofluorescence staining using the DNA dye, Hoechst, to reveal segmented (apoptotic) nuclei; TUNEL (to identify apoptotic nuclei with fragmented DNA); and an anti-*Chlamydia* antibody to identify infected cells. While many apoptotic nuclei were present among uninfected cells, very few cells containing *Chlamydia* inclusions had apoptotic nuclei. Consistent with previous results [22,27], more than 95% of the cells containing *Chlamydia* inclusions were resistant to STS-induced apoptosis (Figure 1).

The LGV/L2 serovar of C. trachomatis produces a transmembrane protein, IncG, which localizes to the inclusion membrane and interacts specifically with 14-3-3β [32]. In uninfected cells in the presence of survival signals, the PI3K pathway leads to phosphorylation and activation of AKT/PKB, and subsequent phosphorylation of BAD, which in its phosphorylated form is sequestered in the cytoplasm by cytosolic 14-3-3 [35,36]. In order to explore the possibility that 14-3-3 recruitment by the *Chlamydia* inclusion may increase resistance to apoptosis, cells that had been infected with the LGV/L2 serovar were incubated with a PI3K inhibitor (LY294002) before treatment with STS. Under conditions in which STS-mediated apoptosis was inhibited more than 95% by C. trachomatis infection, over a third of the infected cells that had been pretreated with the PI3K inhibitor underwent apoptosis (Figure 1).

The PI3K inhibitor was added 24 h prior to treatment with STS (8 h post-infection) (Figure 1) or 6 h prior to treatment with STS (26 h post-infection) (unpublished data). Both protocols gave similar results, suggesting that the PI3K inhibitor has its effect on apoptosis at a post-entry step.

To investigate the specificity of PI3K-dependent resistance to apoptosis, infected cells were treated separately with inhibitors that target components of different anti-apoptotic pathways, such as JNK1/2 (SP600125), P38 (SB203580), mTOR (rapamycin), and synthesis of heat shock proteins (KNK437), including hsp70 [38,39]. Using trypan blue exclusion assays, we verified that the drugs were not toxic to uninfected cells at the concentrations used. With the possible exception of the JNK1/2 inhibitor, which increased slightly the level of STS-induced apoptosis, none of the inhibitors had a significant effect on resistance to apoptosis (Figure 1). Thus, the PI3K pathway has a predominant role in protecting infected cells against STS-mediated apoptosis.

To determine whether the PI3K pathway is involved in protection of C. pneumoniae-infected cells from apoptosis, we measured resistance to STS-induced apoptosis of C. pneumoniae-infected cells in the presence and absence of LY294002 and found no change in the level of protection (unpublished data). Thus, while other mechanisms such as BH3-only protein degradation may provide protection against apoptosis of C. pneumoniae-infected cells, the PI3K pathway does not contribute to resistance. Genome sequencing has revealed an IncG homolog is present in C. trachomatis L2 but not in C. pneumoniae [11,40,41]. Consistent with this finding, the IncG-binding adaptor 14-3-3β is localized on C. trachomatis L2 inclusions but not on C. pneumoniae inclusions [32]. Considered together with our findings, these data indicate that sequestration of 14-3-3β to the inclusion is required for PI3K-dependent protection.

### LY294002 Pretreatment Increases Cytochrome *c* Release in C. trachomatis-Infected Epithelial Cells

**Figure 2:**
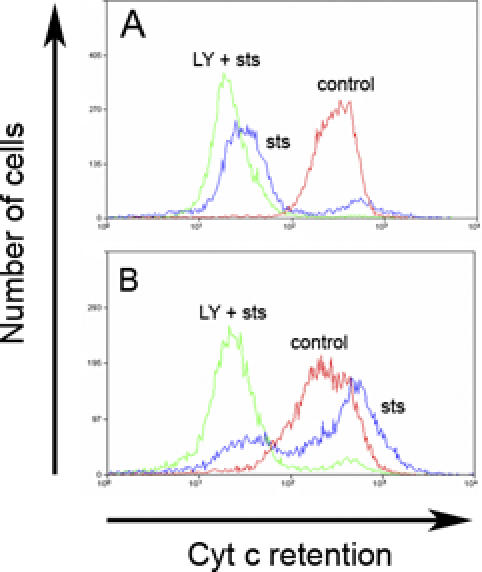
Cytochrome *c* Release from C. trachomatis-Infected Cells Treated with the PI3K Inhibitor Cells were infected with C. trachomatis at an MOI of 1.0 for 26 h (B) or mock-infected (A), and then incubated with control buffer or 50 μM LY294002 (LY) or control buffer for 6 h before treating with 2 μM STS overnight. Cells were then collected for cytofluorimetry, permeabilized with detergent, and incubated with anti-cytochrome *c* antibody, as described in Materials and Methods. Cytochrome *c* was released from uninfected cells that were treated with STS (A), but was retained in infected cells treated with STS (B). However, cytochrome *c* was released in infected cells that had been pre-incubated with LY294002 before STS treatment (B). One experiment of two representative experiments performed on separate days is shown.

The release of cytochrome *c* is a critical step in the mitochondrial pathway to apoptosis, which is triggered by STS. We next tested whether the PI3K-mediated protection was located upstream or downstream of cytochrome *c* release. In the absence of apoptotic stimuli, cytochrome *c* is localized in mitochondria. Typical results, shown in Figure 2A, indicate that less than 10% of uninfected cells retained cytochrome *c* in their mitochondria after treatment with STS. Under the same conditions, more than 60% of STS-treated C. trachomatis-infected cells retained their mitochondrial pool of cytochrome *c* (Figure 2B). However, the percentage of infected cells showing retention of mitochondrial cytochrome *c* decreased significantly when those cells were preincubated with LY294002 before incubation with STS (Figure 2B). These results suggest that PI3K-dependent inhibition of apoptosis in *C. trachomatis-*infected cells is associated with the ability of the cells to maintain an intact mitochondrial membrane. The pro-survival activity of PI3K/AKT is therefore localized upstream of mitochondrial cytochrome *c* release.

### AKT Activation Is Downstream from PI3K Activation during C. trachomatis Infection

In order to further elucidate the anti-apoptotic mechanisms induced by infection, we measured the phosphorylation status of an essential survival-signaling mediator that is downstream from PI3K, the serine/threonine kinase, AKT/PKB. In several cell lines, PI3K phosphorylates and thereby activates AKT, which then promotes survival through phosphorylation of multiple targets [35,36]. We thus measured phosphorylation by immunoblots using antibodies against a phosphorylation site of AKT (residue Ser^473^) that leads to AKT activation in epithelial cells under different conditions.

**Figure 3:**
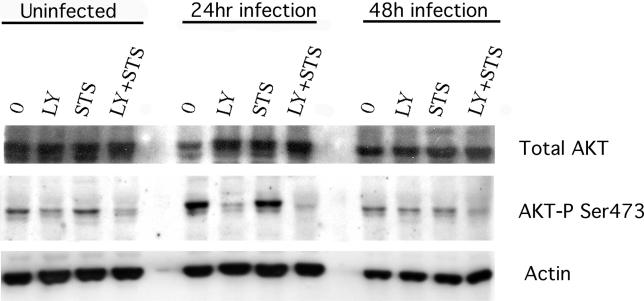
AKT Phosphorylation in Cells during Apoptosis of C. trachomatis-Infected Cells Cells were infected with C. trachomatis at an MOI of 1.0 for 24 or 48 h, or mock-infected, and then incubated with 50 μM LY294002 (LY) or control buffer for 6 h before treating with control buffer or 2 μM STS overnight. Cells were then collected for Western immunoblotting and analyzed for phosphorylation of AKT on residue Ser^473^, as described in Materials and Methods. STS treatment by itself did not affect AKT Ser^473^ phosphorylation in infected cells, but LY294002 caused AKT Ser^473^ to become partially dephosphorylated. This residue became dephosphorylated almost completely when LY294002 was combined with STS in infected cells. One experiment of two representative experiments performed on separate days is shown.

AKT phosphorylation/activation increased after a 24-h infection with *C. trachomatis,* and decreased back to basal levels by 48 h after infection (Figure 3). STS treatment did not affect the AKT phosphorylation level in uninfected or infected cells. In cells infected for 24 h that were then preincubated for 6 h with LY294002 (before incubation with STS or control buffer overnight), essentially all the AKT became dephosphorylated (Figure 3). These results suggest that *Chlamydia* infection leads to PI3K activation, which is essential for subsequent phosphorylation and activation of the downstream effector, AKT.

**Figure 4:**
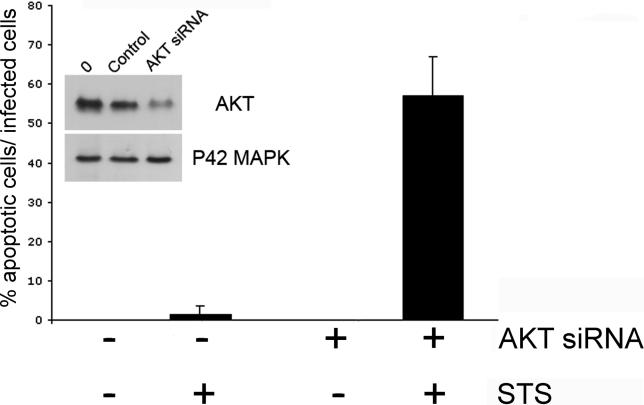
Effect of Inhibition of AKT Expression on Apoptosis of C. trachomatis-Infected Cells Cells were transfected with AKT siRNA (+) or control reagents (−) for 8 h, infected with C. trachomatis at an MOI of 1.0 for 32 h, and then incubated with 2 μM STS or control buffer overnight. The efficiency of transfection with AKT siRNA in epithelial cells was monitored by co-transfecting with fluorescein-labeled irrelevant siRNA, which showed that approximately 80% of cells were transfected during the experiments (unpublished data). DNA was revealed with Hoechst and *Chlamydia* with anti-*Chlamydia* antibody. The extent of nuclear condensation was measured only in cells that contained *Chlamydia* vacuoles. No nuclear condensation was observed in infected cells that expressed normal levels of AKT, but STS led to nuclear condensation in infected cells that had been transfected with AKT siRNA. One experiment of two representative experiments performed on separate days is shown. *Inset:* Inhibition of AKT expression in epithelial cells by RNA interference. Cells were transfected with siRNA for AKT or controls. After 48 h, the cells were harvested and AKT protein levels were measured by Western immunoblotting. The control reagents did not affect AKT expression, and AKT siRNA had no effect on the expression of another protein, the P42 MAP kinase.

In order to rule out the possibility that the PI3K inhibitor, LY294002, may have nonspecific effects and to confirm a role for AKT in the resistance to apoptosis, we inhibited AKT expression specifically in the epithelial cells by RNA interference. 48 h after transfection with AKT siRNA, more than 70% of the protein levels of AKT were reduced in uninfected cells, as verified by Western blotting (Figure 4, inset); and we verified that AKT siRNA by itself did not induce apoptosis of uninfected cells (unpublished data). The epithelial cells were first transfected with AKT siRNA, infected with *C. trachomatis,* and then treated with STS. In untransfected cells that contained chlamydial inclusions, very few were sensitive to STS-induced apoptosis determined by fluorescence microscopic examination for nuclear condensation. In contrast, more than half of the AKT siRNA transfected cells that contained chlamydial inclusions exhibited apoptosis after treatment with STS (Figure 4). Taken together, these results suggest that AKT plays a major role in protection against apoptosis of *Chlamydia*-infected cells.

### PI3K Activation Leads to BAD Phosphorylation in Epithelial Cells Infected with C. trachomatis

AKT has been shown to phosphorylate BAD on residue Ser^136^, which causes BAD to lose its pro-apoptotic activity [35,36]. In order to determine whether phosphorylated BAD could play a role in resistance to apoptosis during C. trachomatis infection, we increased BAD protein-expression by transfecting epithelial cells with a BAD eukaryotic expression plasmid prior to infection and observing the phosphorylation of BAD by Western blotting. Although BAD was expressed at high levels in both infected and uninfected cells, the sensitivity to STS-induced apoptosis of the cells was similar to the sensitivity of cells expressing normal levels of BAD (unpublished data).

**Figure 5:**
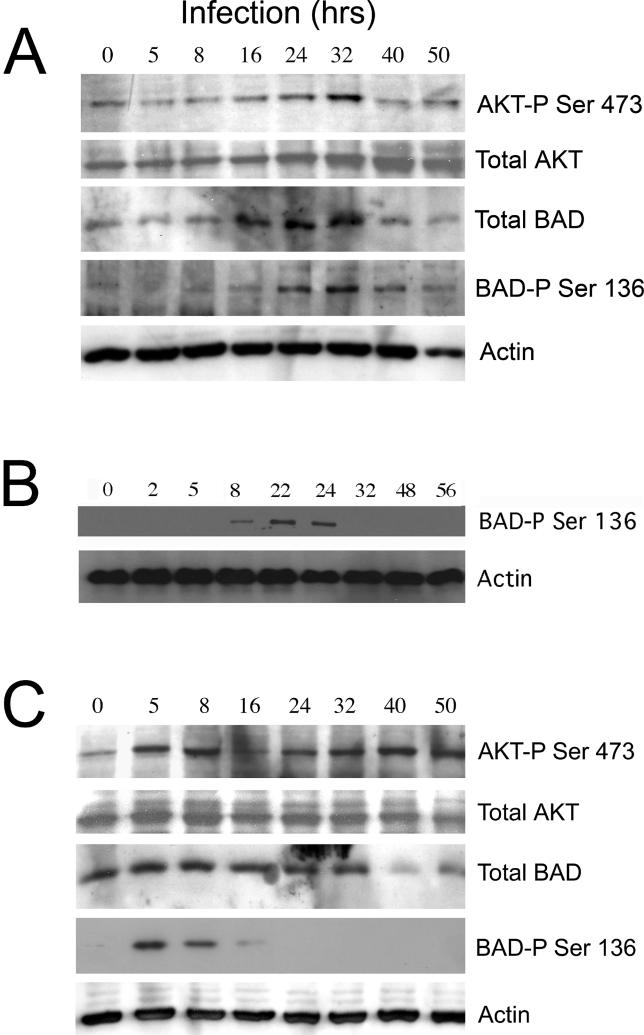
Time Course of BAD Phosphorylation during C. trachomatis or C. muridarum Infection (A) Epithelial cells were transfected with a BAD expression plasmid prior to infection, as described in Materials and Methods. Cells over-expressing BAD were infected with C. trachomatis at an MOI of 1.0 for the indicated times. Cells were then collected for Western immunoblotting and analyzed for AKT phosphorylation (top row), total AKT protein (second row), total BAD protein (third row), phosphorylation of BAD on residue Ser^136^ (fourth row), or actin (bottom row), as described in Materials and Methods. *Chlamydia* infection at this MOI led to a decrease in the level of total BAD protein after 32 h of infection. For AKT residue Ser^473^, there was a low level of phosphorylation in uninfected cells (time 0), but there was a noticeable increase in phosphorylation for both AKT and BAD after 16 h of infection. Phosphorylation levels decreased after 32 h of infection for both BAD and AKT. *Chlamydia* infection had no effect on actin protein levels (bottom row). One experiment of three representative experiments performed on separate days is shown. (B) Cells expressing endogenous levels of BAD were infected with C. trachomatis at an MOI of 1.0 for the indicated times. Cells were then collected for Western immunoblotting and analyzed for phosphorylation of BAD on residue Ser^136^ (top row) or actin (bottom row). The time course of BAD phosphorylation in cells expressing only endogenous BAD was similar to the time course in cells that had been transfected with the BAD expression plasmid (A). One experiment of two representative experiments performed on separate days is shown. (C) Cells over-expressing BAD were infected with C. muridarum at an MOI of 1.0 for the indicated times. Cells were then collected for Western immunoblotting and analyzed for AKT phosphorylation (top row), total AKT protein (second row), total BAD protein (third row), phosphorylation of BAD on residue Ser^136^ (fourth row), or actin (bottom row). *Chlamydia* infection at this low MOI led to a decrease in the level of total BAD protein after 32 h of infection. BAD was phosphorylated significantly within 5 h of infection, but phosphorylation levels decreased by 8 h post-infection and were not measurable after 1 d of infection. One experiment of three representative experiments performed on separate days is shown.

The total amount of BAD decreased in infected cells after 32 h of infection (Figure 5A), consistent with our previous results obtained at a higher multiplicity of infection (MOI) with cells that expressed only endogenous BAD [27,28]. There was little basal phosphorylation in uninfected cells, but BAD phosphorylation increased 8 h post-infection, peaked between 24–32 h post-infection, and decreased thereafter (Figure 5A). A similar time course for BAD phosphorylation was observed in infected cells expressing only endogenous BAD (Figure 5B), suggesting that the total level of BAD protein does not affect the kinetics of BAD phosphorylation.

IncG is also expressed by the genome of *Chylamdia muridarum,* and 14-3-3β is recruited to C. muridarum inclusions [32]. However, C. muridarum infection protects the host cell against apoptosis poorly, if at all [42,43]. To investigate whether BAD might interact with inclusion-bound 14-3-3β in C. muridarum-infected cells, cells over-expressing BAD were infected with C. muridarum and the level of AKT and BAD phosphorylation was measured (Figure 5C). There may be an increase in the extent of AKT phosphorylation in infected cells, but the level of BAD phosphorylation peaked within 5 h of infection and decreased by 8 h after infection (Figure 5C). Almost no phosphorylated BAD remained by 16 h of infection. Thus, no phosphorylated BAD remains in cells infected with C. muridarum at times when cells infected with C. trachomatis and C. pneumoniae begin to exhibit resistance to apoptosis [22,25,27], suggesting that rapid dephosphorylation of BAD may account for the poor protection against apoptosis conferred by C. muridarum infection.

**Figure 6:**
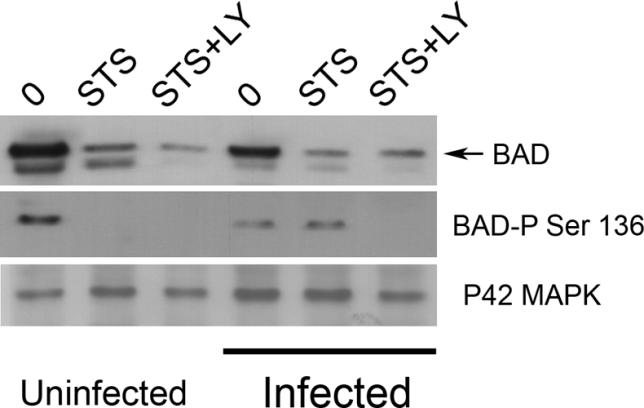
Effect of PI3K on BAD Phosphorylation during C. trachomatis Infection Cells over-expressing BAD were infected with C. trachomatis at an MOI of 1.0 for 26 h or mock-infected, and then incubated with 50 μM LY294002 (LY) or control buffer for 6 h before treating with 2 μM STS overnight. Cells were then collected for Western immunoblotting and analyzed for total BAD protein (top row), phosphorylation of BAD on residue Ser^136^ (middle row), or total P42 MAP kinase protein (bottom row), as described in Materials and Methods. C. trachomatis infection led to a decrease in the level of BAD, which was further decreased by STS treatment. Pre-incubation with LY294002 did not further alter the level of total BAD (top row). STS-treatment of uninfected cells caused complete dephosphorylation of BAD, but BAD remained phosphorylated after STS-treatment of infected cells (middle row). However, pre-incubation of infected cells with LY294002 before treatment with STS resulted in complete dephosphorylation of BAD (middle row). Infection had no effect on total P42 MAP kinase protein levels (bottom row). One experiment of three representative experiments performed on separate days is shown.

The potential role of the PI3K pathway in activating BAD in cells infected with C. trachomatis was investigated by Western blot. Phosphorylated BAD was detected in both infected and uninfected cells, but disappeared after STS treatment of uninfected cells (Figure 6). Moreover, the levels of total BAD decreased with STS treatment in both uninfected and infected cells, consistent with previous studies showing cleavage of BAD during cell death [44]. Conversely, the level of phosphorylated BAD remained high in infected cells treated with STS, unless the cells were pretreated with LY294002 (Figure 6). Thus, infection inhibits BAD dephosphorylation, which is downstream from PI3K/AKT activation.

We have recently reported that C. trachomatis infection leads to degradation of several pro-apoptotic BH3-only proteins [27,28]. In order to determine if inhibition of PI3K by LY294002 specifically affected degradation of BH3-only proteins, we infected epithelial cells with *C. trachomatis. C. trachomatis* infection resulted in degradation of both BIM and BAD, but the PI3K inhibitor had no effect on infection-induced degradation of either BIM or BAD (unpublished data), suggesting that phosphorylation of BAD may be acting as an alternate anti-apoptotic pathway, in parallel with induction of BH3-only protein degradation.

### Infection with C. trachomatis Causes Phosphorylated BAD to Become Localized on Chlamydial Inclusions

**Figure 7:**
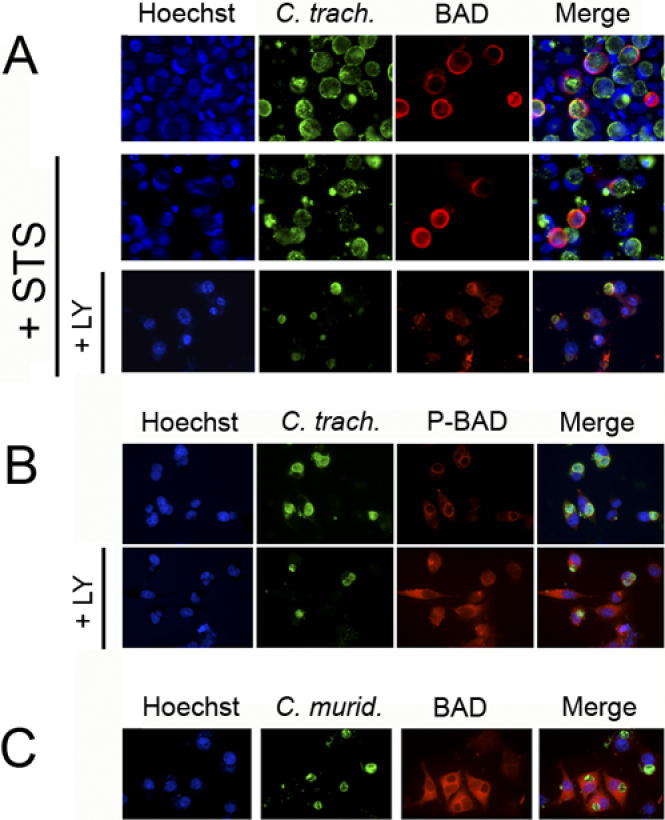
Localization of BAD and Phosphorylated BAD in Cells Infected with C. trachomatis or C. muridarum (A) In order to visualize BAD by immunofluorescence, epithelial cells were transfected with the BAD expression plasmid prior to infection. Cells over-expressing BAD were infected with C. trachomatis for 26 h and then incubated with 50 μM LY294002 (LY) or control buffer for 6 h before treating with 2 μM STS or control buffer overnight. BAD is localized to the surface of *Chlamydia* vacuoles in infected cells, with or without STS treatment (first and second rows). The co-localization of BAD and *Chlamydia* vacuoles is lost in STS-treated infected cells that had been pre-incubated with LY294002 (bottom row). (B) Cells over-expressing BAD were infected with C. trachomatis for 15 h and then incubated with control buffer (top row) or 50 μM LY294002 (LY) (bottom row) for 6 h. Phosphorylated BAD is concentrated around the early *Chlamydia* vacuoles, but has a cytosolic distribution in infected cells after treatment with LY294002. Cells treated with LY294002 were exposed at a longer time in order to visualize background phosphorylated-BAD staining. (C) Cells over-expressing BAD were infected with C. muridarum for 15 h. BAD has a cytosolic distribution in infected cells, even in the absence of LY294002. DNA was revealed with Hoechst (blue), *Chlamydia* with FITC-conjugated anti-*Chlamydia* antibody (green), and BAD with Texas Red-conjugated anti-BAD antibody or phosphorylated BAD with Texas Red-conjugated antibody recognizing BAD phosphorylated on residue Ser^136^ (red). In each case, one experiment of two representative experiments performed on separate days is shown.

In the absence of apoptotic stimuli, BAD is uniformly distributed in the cytosol (unpublished data). However, in infected cells, most BAD is localized to the *Chlamydia* inclusion, in the presence or absence of STS, suggesting that the pro-apoptotic protein is sequestered by the inclusion (Figure 7A, first and second rows). Incubation of infected cells with LY294002 prior to STS treatment resulted in a decrease in BAD associated with the *Chlamydia* inclusion, which coincided with nuclear condensation in infected cells (Figure 7A, third row).

The adapter protein 14-3-3 binds only to BAD that is phosphorylated [35,36]. To determine whether phosphorylated BAD is recruited by the *Chlamydia* vacuole, cells over-expressing BAD were infected with *C. trachomatis,* and phosphorylated BAD was localized by immunofluorescence using antibodies that recognize BAD phosphorylated on residue Ser^136^. To exclude the possibility of artifactual co-localization between phosphorylated BAD and chlamydiae due to the large size of mature inclusions, cells were infected for only 15 h. Figure 7B (top row) shows that there is co-localization between phosphorylated BAD and the early chlamydial inclusion. Similar results were found in cells infected for longer times (unpublished data). In order to examine whether phosphorylation of BAD is required for the co-localization, the infected cells were also treated with the PI3K inhibitor, LY294002. Longer exposures failed to reveal co-localization between the inclusion and remaining phosphorylated BAD (Figure 7B, bottom row).

Given that BAD was quickly dephosphorylated during infection with *C. muridarum,* we also measured the localization of BAD in cells over-expressing BAD that were infected with *C. muridarum.* Cells were infected with C. muridarum for 15 h, at which time little BAD was still phosphorylated. There was no co-localization between BAD and the early inclusion (Figure 7C), although similar results were also obtained with larger inclusions (unpublished data). The PI3K inhibitor, LY294002, had no effect on the distribution of BAD in C. muridarum-infected cells (unpublished data).

**Figure 8:**
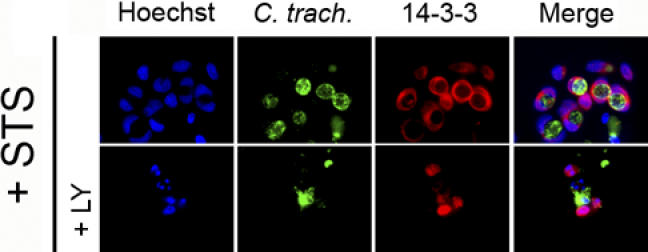
Localization of 14-3-3β in C. trachomatis-Infected Cells Cells expressing endogenous levels of BAD were infected with C. trachomatis at an MOI of 1.0 for 26 h, and then incubated with 50 μM LY294002 (LY) or control buffer for 6 h before treating with 2 μM STS overnight. DNA was revealed with Hoechst (blue), *Chlamydia* with FITC-conjugated anti-*Chlamydia* antibody (green), and 14-3-3 with Texas Red-conjugated anti-14-3-3 antibody (red), as described in Materials and Methods. 14-3-3 is localized primarily on the *Chlamydia* vacuole in infected cells, even in the presence of STS (top row). The co-localization of 14-3-3 and the *Chlamydia* vacuole is lost in infected cells that had been pre-incubated with LY294002 before STS treatment (bottom row). One experiment of three representative experiments performed on separate days is shown.

Since BAD phosphorylation leads to sequestration of BAD by cytosolic 14-3-3 in uninfected cells [35], and the 14-3-3β protein has been previously shown to localize on the surface of the C. trachomatis vacuole in infected cells [32], we examined the distribution of 14-3-3β in infected and uninfected cells, with or without STS treatment. As expected, 14-3-3β was distributed uniformly in uninfected cells (unpublished data). During C. trachomatis infection, 14-3-3β was localized in a ring around the inclusion, in agreement with previous results [32] (unpublished data). Interestingly, 14-3-3β remained localized in the ring despite treatment with STS (Figure 8, top row). But 14-3-3β distributed throughout infected cells that had been pretreated with LY294002 before incubation with STS (Figure 8, bottom row), which could be due in part to condensation of the dying host-cell and fragmentation of the inclusion. Thus, protection of C. trachomatis-infected cells against apoptosis is due at least partly to activation of PI3K and AKT, which correlate with BAD phosphorylation and the sequestration of phosphorylated BAD by 14-3-3β on the surface of the *Chlamydia* inclusion.

## Discussion

We demonstrate here that the PI3K pathway plays a prominent role in protecting C. trachomatis-infected cells from apoptosis. Among different signaling pathways considered, only inhibition of the PI3K pathway removed the protection provided by infection against apoptosis. Inhibitors targeting JNK1/2, P38, mTOR or synthesis of heat shock proteins were unable to reverse protection against apoptosis, although the JNK1/2 inhibitor had a slight effect.

**Figure 9:**
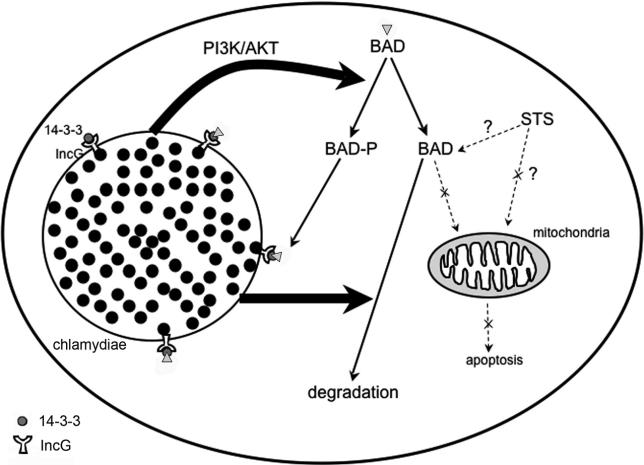
Summary of the Effects of C. trachomatis Infection on BAD Phosphorylation, BAD Degradation, and Host-Cell Death Infection with C. trachomatis leads to activation of PI3K, which in turns results in AKT activation and subsequent BAD phosphorylation. In parallel, infection causes degradation of most unphosphorylated BAD. Simultaneous expression of IncG on the chlamydial inclusion and binding of 14-3-3β by IncG allows the inclusion to recruit phosphorylated BAD, preventing the ability of the remaining BAD to translocate to the mitochondria and induce host-cell death.

The PI3K pathway leads to phosphorylation of AKT, which in C. trachomatis-infected cells remains phosphorylated even after treatment with STS. Consistent with a role for this pathway in resistance to apoptosis, inhibition of PI3K or depletion of AKT causes STS-treated infected cells to die through apoptosis, through a mechanism involving cytochrome *c* release. AKT activation also results in BAD phosphorylation, which can be blocked in infected cells by incubation with the PI3K inhibitor, and with a time course that is consistent with the appearance of protection against apoptosis of cells infected with C. trachomatis [22]. BAD co-localizes with the adapter protein, 14-3-3β, on the surface of the chlamydial inclusion membrane even in infected cells that had been treated with STS. As 14-3-3β co-localizes with the chlamydial protein IncG on the surface of the inclusion [32], these results suggest that this IncG-14-3-3β interaction allows the inclusion to sequester BAD away from mitochondria, where it could stimulate release of cytochrome *c.* Simultaneous activation of the PI3K pathway during infection maintains BAD in its phosphorylated state (Figure 9).

A role for IncG in PI3K-dependent protection against apoptosis is suggested by the observation that the PI3K inhibitor removed protection in cells infected with *C. trachomatis,* but not in cells infected with *C. pneumoniae,* which does not express the IncG protein. Phosphorylation of BAD also appears to be necessary for co-localization with the inclusion, since BAD does not co-localize with C. muridarum inclusions at a time when little BAD is phosphorylated, even though C. muridarum expresses IncG [32].

For safe measure, *Chlamydia* infection also leads to degradation of most of the BAD protein [27,28], whose degradation is measurable after 1 d of infection (Figure 9). Any remaining, but presumably functional, BAD could be sequestered away from mitochondria by 14-3-3β, via the chlamydial protein IncG, whose expression begins within 2 h after internalization of the bacteria by host cells [32]. IncG is also phosphorylated in infected cells, and the phosphorylation is required for IncG interaction with 14-3-3β [32]. Further exploration of the cross talk between chlamydial inclusion membrane proteins and host-cell signaling pathways will likely yield valuable new information on *Chlamydia*-dependent modulation of host-cell biology.

It is still unknown what mechanism could lead to PI3K activation in C. trachomatis-infected cells. An obvious possibility would be that chlamydiae secrete anti-apoptotic factors through their type III secretion apparatus. Secreted bacterial proteins of other pathogens can stimulate or interfere with specific host-cellular processes for the benefit of the pathogen [48]. In particular, bacterial proteins secreted through the type III secretion apparatus by a number of pathogens have already been shown to modulate cell death during infection [49–53]. Thus, Salmonella enterica protects infected epithelial cells from apoptosis through secretion of SopB, which induces sustained activation of AKT [53].

Alternatively or concurrently, a host-cell stress response could result in PI3K activation. Signaling through AKT is known to play a key role in protection of cells from a variety of toxic stresses [38,54–58], as may occur during infection by intracellular pathogens. In the specific case of *Chlamydia,* infection is known to increase mitochondrial metabolism and oxidative stress [59–61]. In cells exposed to oxidative stress, down regulation of the PI3K/AKT pathway results in higher levels of apoptosis [62] and oxidants stimulate the AKT survival pathway [63,64]. Interestingly, mechanical stress, which could be caused by the presence of a growing inclusion, induces rapid phosphorylation of AKT in various cell types [65,66]. We thus propose that *Chlamydia* could secrete specific, as-yet-unidentified proteins that could activate the PI3K pathway directly, or that oxidative or mechanical stress induced by infection leads to activation of the host cell PI3K/AKT pathway, resulting in protection from apoptosis.

## Materials and Methods

### Cells, bacteria, and chemical reagents.

HeLa 299 cells (ATCC, American Type Culture Collection, Manassas, Virginia, United States) were cultured in a humidified incubator at 37 °C with 5% CO_2_ in Dulbecco's modified Eagle medium (DMEM) supplemented with glutamax-1 (Life Technologies Incorporated, Rockville, Maryland, United States), 10% heat-inactivated fetal calf serum, and 25 μg/ml gentamycin. The LGV/L2 strain of C. trachomatis and the TW-183 strain of C. pneumoniae were from the ATCC. The “mouse pneumonitis” strain (MoPn), also known as *C. muridarum,* was a kind gift from Dr. Roger Rank (University of Arkansas, Little Rock, Arkansas, United States). The number of bacterial inclusion forming units was determined using an immunofluorescence method as previously described [27,67]. The different signaling pathway inhibitors used in this study (SP600125, SB203580, rapamycin, and KNK37) were from Calbiochem (La Jolla, California, United States), except for LY294002, which was from Sigma (St. Louis, Missouri, United States).

### Cell culture and infection.

HeLa cells growing at 70% confluence on tissue culture flasks (Costar) were infected with the LGV/L2 strain of *C. trachomatis, C. muridarum,* or C. pneumoniae at an MOI of 1.0 and incubated for the indicated times in an incubator at 37 °C under 5% CO_2_. Under these conditions, > 80% of the cells contained chlamydial vacuoles after 1 d of infection. In order to induce apoptosis, infected and control uninfected cells were treated overnight with 2 μM STS (Sigma). When indicated, cells were incubated with LY294002 before STS treatment. Both adherent cells and cells in suspension were collected for either flow cytometric or Western blot analysis, while only adherent cells were fixed for fluorescence microscopy.

### Transfections.

As BAD is not visible by immunofluorescence in cells expressing endogenous levels of BAD, BAD was over-expressed where indicated. The BAD plasmid was transfected using Lipofectamine Plus reagent (Invitrogen Life Technology, Carlsbad, California, United States) following manufacturer's instructions. Briefly, 12 h after trypsinization and seeding into 24-well plates, HeLa cells were incubated for 4 h at 37 °C with 250 μl of DMEM containing 400 ng of pEBG-mBAD (Cell Signaling Technology, Beverly, Massachusetts, United States), 1 μl of Lipofectamine, and 4 μl of Plus reagent. Serum-containing medium was added into each well, and cells were incubated for up to 48 h.

Transfection of siRNA for AKT was performed as follows: HeLa cells were plated in 24 well-plates for 12 h before transfection in order to reach 70% confluence on the following day, when the medium was replaced by 0.25 ml of serum-containing medium. The siRNA complex was formed immediately before transfection by adding 2.5 μl of the TransIT-TKO transfection reagent (Mirus Corporation, Madison, Wisconsin, United States) drop-wise into 50 μl of serum-free RPMI 1640 (Invitrogen Life Technology). After gentle mixing, the incubation was performed for 10 min at room temperature. 50 nM siRNA AKT (final concentration) was added to the diluted transfection reagent, mixed gently, and incubated for 10 min at room temperature. Finally, 50 μl of this mixture was added to each well, the plate was rocked gently, and further incubated for 48 h at 37 °C. The sequences of the siRNA AKT were: sense, 5′-UGCCCUUCUACAACCAGGATT; antisense, 5′-UCCUGGUUGUAGAAGGGCATT.

For pEGB m-BAD- or siRNA AKT-transfected and infected cells, the infection with *Chlamydia* was begun 8 h after transfection. As a negative control for siRNA AKT, cells were transfected with the same amount of irrelevant RNA, before infection with *Chlamydia*.

### Flow cytometric analysis of cytochrome *c* release.

HeLa cells were harvested with PBS containing 1% trypsin and 1 mM EDTA, and then treated with digitonin (200 μg/ml) (Sigma) in PBS for 10 min on ice in order to release cytosolic cytochrome *c*. Cells were washed with three equivalent volumes of PBS containing 0.3% BSA, centrifuged (1,000 g, 10 min, 4 °C), and fixed in 4% neutral-buffered paraformaldehyde for 20 min at room temperature. Cells were then washed again in PBS and incubated in blocking buffer (3% BSA, 0.1% Triton X-100 in PBS) for 1 h. Finally, cells were incubated overnight at 4 °C with a 1:500 dilution of anti-cytochrome *c* monoclonal antibody (clone 6H2B4, BD Pharmingen, San Diego, United States). On the following day, cells were washed three times with blocking buffer, and incubated for 1 h with an FITC-conjugated goat anti-mouse antibody (Caltag Laboratories, Burlingame, California, United States). The cells were washed three times in PBS and analyzed by flow cytometry (FL1), as previously described [68].

### Detection of apoptotic nuclei by Hoechst or TUNEL staining.

HeLa cells were grown on glass slides in 24-well plates, washed with PBS, and fixed in 4% neutral-buffered paraformaldehyde for 30 min. DNA strand breaks were identified using a terminal deoxynucleotidyltransferase-mediated dUTP nick end labeling (TUNEL) assay kit, following the manufacturer's protocol (Roche Applied Science, Basel, Switzerland), as previously described [69]. In order to detect infected and apoptotic cells simultaneously, slides were counterstained using an anti-*Chlamydia* genus monoclonal antibody (clone C5/C8, Argene Biosoft, Varilhes, France) at 1:400 dilution, and a Texas Red-conjugated or FITC-conjugated goat anti-mouse polyclonal antibody (1:200 dilution) plus Hoechst (1 μg/ml) in PBS. Slides were mounted in antifade medium (Dako, Glostrup, Denmark) and examined using an epifluorescence microscope (Zeiss Axioskope) equipped with band pass optical filter sets appropriate for TRITC, FITC, and DAPI dyes. The images were captured by multiple exposures using a cooled CCD camera controlled by Metamorph software.

Cells were also stained with 1 μg/ml Hoechst, which detects both host-cell and chlamydial DNA. In order to quantify the number of apoptotic or non-apoptotic nuclei in both infected and uninfected cells in each cell population, cells were counted in each microscope field, and the average and standard deviation per condition was calculated for the % of apoptotic cells with co-localizing inclusions in at least ten fields, containing an average of 100 cells per field.

### Localization of BAD, phosphorylated BAD, and 14-3-3β by immunofluorescence.

BAD localization was detected on adherent cells using rabbit anti-BAD antibody (Cell Signaling Technology) at 1:50 dilution, as described in the manufacturer's protocol. Phosphorylated BAD was detected with an antibody recognizing BAD phosphorylated on Ser^136^ (Santa Cruz Biotechnology, Santa Cruz, California, United States). 14-3-3β was detected using a mouse anti-14-3-3 antibody directly coupled to TRITC (clone C20; Santa Cruz Biotechnology) diluted at 1:200 in PBS containing 3% BSA. Cells were subsequently counterstained with *Chlamydia* antibody and Hoechst (1 μg/ml), as described above.

### Western blot analysis.

Infected or uninfected cells were seeded in 12-well plates (Costar). Following transfection with either pEBG-mBAD or AKT siRNA, or treatment with LY294002 and/or STS, adherent cells and cells in suspension were pooled and washed in cold PBS. Samples were lysed in Laemmli buffer containing both protease (5 μl/ml, Sigma) and phosphatase inhibitor cocktails (5 μl/ml, Sigma) before storing at −80 °C. Proteins were resolved on 12% SDS-PAGE and transferred onto nitrocellulose membranes (Amersham Biosciences, Little Chalfont, United Kingdom). After blocking in Tris-buffered saline containing 0.05% Tween 20 (TBST) and 5% BSA, the membranes were washed extensively in TBST and immunostained with the following first rabbit polyclonal antibodies diluted in blocking buffer: anti-AKT antibody (1:1000) (from Cell Signaling Technology), anti-AKT phosphorylated on Ser^473^ (1:1000) (Cell Signaling Technology), anti-BAD (1:1000) (Cell Signaling Technology), anti-BAD phosphorylated on Ser^136^ (1:500) (Cell Signaling Technology), anti-BIM (Sigma), anti-tubulin (Sigma), or anti-p42/44 MAPK (1:1000) (Cell Signaling Technology). Following washes with TBST, membranes were incubated with an anti-rabbit polyclonal antibody conjugated to HRP diluted at 1:10000 (Amersham Biosciences). Specific bands were visualized by enhanced chemiluminescence (Amersham Biosciences).

### Statistics.

Data are presented as the mean ± standard deviation of *“n”* experiments. The statistical difference was determined using the paired Student's *t* test. A *p* value of less than 0.05 was considered statistically significant.

## Abbreviations

LGV: lymphogranuloma venereum
MAP kinase: mitogen-activated protein
MOI: multiplicity of infection
P13K: phosphatidylinositol-3 kinase
PKB: protein kinase B
STS: staurosporine
